# Failure energy evolution of coal–rock combination with different inclinations

**DOI:** 10.1038/s41598-022-23813-6

**Published:** 2022-11-14

**Authors:** Yongjiang Yu, Jingjing Liu, Yuntao Yang, Pengbo Wang, Zhenmeng Wang, Zhiyuan Song, Jiaming Liu, Shangqing Zhao

**Affiliations:** 1 College of Mining Engineering, Liaoning Technical University, Fuxin, Liaoning, 123000 China; 2 Gas Research Branch, China Coal Technology and Engineering Group, Chongqing Research Institute, Chongqing, 400037 China

**Keywords:** Energy science and technology, Engineering

## Abstract

In this paper, in the deformation and damage process under different confining pressures, the energy evolution characteristics and damage mechanism of coal–rock combinations with different inclination angles are studied. Based on the brittleness indexes of coal rock combinations, the evolution rules between brittleness indexes and the inclination are explored, as well as the confining pressure of coal rock combinations; then, the influence mechanism of the inclination angle of coal rock combinations on the plastic yielding degree, energy dissipation level, crack extension and fracture speed in the pre-peak stage is revealed. The composite specimens are mainly damaged due to oblique shear and accompanied by tensile damage; In the deformation and damage, various energies of coal rock composites are distributed as a negative exponential function of the inclination angle, which is significantly affected by the change of the confining pressure.

## Introduction

With the gradual increase in the depth of coal mining, many corresponding disaster problems have been focused on. According to a large number of geological accident investigations, it is shown that most mine disasters are caused by the disaster destabilization of the mechanical system composed of coal–rock rather than the damage of individual rock or coal bodies. The damage to coal–rock combination is not only affected by the stress state and fracture structure surface but also related to the structural form. In the process of coal–rock combination failure, energy plays a fundamental role, and the coal–rock combination failure process is the essential result of the sudden release of energy. In the failure process of coal–rock combination, the evolutionary characteristics of energy are closely related to the structural form of coal–rock combination. Therefore, it is important to study the energy evolution characteristics and damage mechanism of different coal–rock combinations in the process of damage for disaster prevention and mitigation in deep mines.

At present, researchers have conducted studies on the deformation and strength characteristics of intact coal or rock bodies and coal–rock combinations under a three-way stress state^1–3^. Zhan et al.^4^ defined and derived the relative physical and mechanical parameters of coal–rock combinations (CRCB) to analyze the effects of coal–rock height ratio (CRHR), coal–rock body behavior and interface parameters on the CRCB. By using the RMT rock mechanics test system and acoustic emission real-time monitoring system to combine the evolution laws of fractal and acoustic emission signals, Yang et al.^5^ conducted uniaxial compression tests on coal, sandstone, and three composite specimens and investigated the crack extension and damage mechanism of the composite specimens. Based on uniaxial loading and uniaxial cyclic loading tests, Bao Pan et al.^6^ studied the deformation characteristics and loading damage laws of coal–rock combinations under different coal–rock height ratios. To analyze the deformation and strength characteristics of different coal–rock combinations and discuss their acoustic emission behaviors, Du and Wang et al.^7^ conducted conventional triaxial compression tests on gas-bearing coal, gas-bearing coal mudstone combination, and gas-bearing coal sandstone combination using RLW-500G triaxial test system, and unloading tests on gas-bearing coal sandstone combination specimens. Liu et al.^8^ investigated the effects of fracture length and angle on the mechanical properties and damage modes of the combined rock coal seams through uniaxial compression tests on 21 sets of combined rock coal specimens containing cracks. In order to investigate the influence of combination mode on the mechanical properties and damage characteristics of coal–rock combinations, Zhang et al.^9^ conducted uniaxial compression and triaxial compression test studies on rock-coal–rock, rock-coal and coal–rock combination specimens using the MTS815 rock mechanics system. The test results showed that the damage of the combination specimens was mainly concentrated in the coal body part. By using the MTS815 test machine, Zuo et al.^10^ also verified that the weak coal interlayer changed the overall damage form of coal in the rock-coal–rock combination and reduced the overall stability of the coal body. Guo et al.^11^ conducted a uniaxial compression test study and also conducted PFC2D systematic numerical simulations to explore the influence of coal seams on the mass mechanical behavior and damage characteristics of rock-coal–rock composites (RCR); at the same time, it was found that the deformation and strength behavior of RCR specimens depends on both the thickness and the confining pressure of coal.

Since it is difficult to study rock engineering problems from a macroscopic perspective, various rock engineering problems have been studied from an energy perspective in recent years, including rock explosion, impact ground pressure, coal column destabilization, roadway support, and bulk material vibration^12–14^. To make its application more scientific and promising, the evolution law of energy within the rock under different loading methods must be clarified first, which is also the basic content of the rock energy principle. Focusing on the mechanical properties, permeability, and energy properties of coal seam tuffs in coal penetration tunnels, Zhao and Liu et al.^15^ conducted a triaxial compression and permeability test on tunnel coal seam tuffs using the MTS815 rock mechanics system and acoustic emission technique; they found that gas pressure and confining pressure have a significant effect on the mechanical properties and permeability of tunnel coal seam tuffs, and gas pressure and confining pressure have significant effects on the mechanical properties and permeability of the tunnel coal bed tuff. Using a split Hopkinson pressure bar (SHPB) device, Gong et al.^16^ analyzed the stress-strain curves, dynamic peak stress-strain, elastic modulus and energy distribution laws of coal–rock combinations at different loading rates; they found that in the high loading rate range, the dynamic stress-strain curves of coal–rock combinations have bimodal characteristics and can be divided into the initial pressure-bearing phase, pressure-decreasing phase, pressure-enhancing phase and destabilization phase; besides, the absorbed energy ratio at different loading rates has a good linear relationship with the incident energy. F. Giuseppe^17^ conducted compression tests on a large number of concrete specimens of different sizes and found that the size effect of energy dissipation density was significantly higher than the uniaxial compressive strength. Zhang and Lu et al.^18^ conducted uniaxial compression and Brazilian splitting tests on coal–rock combinations and established the corresponding numerical models using UDEC triangulation to determine the number, length and macroscopic area of cracks during failure and destabilization; by analyzing the two-dimensional dynamic and static combined loading tests of crack development and energy evolution laws, as well as their strain energy evolution laws, they also found that the larger the height ratio of coal–rock, the higher the accumulated energy and the faster the energy dissipation rate. Wang and Cui^19^ studied the mechanism of energy evolution of sandstone under different circumferential pressures by uniaxial compression tests on two sandstones and obtained fine mechanical parameters using PFC numerical analysis software and Fish program and found that the yield stage of sandstone gradually increased with the increase of circumferential pressure, and the ultimate value of elastic strain energy increased with a good linear variation relationship. Su and Zhang^20^ conducted uniaxial compression tests on marble specimens at different envelope pressures after yielding in axial compression and then performed uniaxial compression tests on damaged specimens; it is shown that less energy was consumed before yielding, the more energy was required for the plastic deformation process during triaxial compression of the specimens, and plastic deformation and energy consumption had good linear characteristics, and more energy was required to make the specimens completely damaged at high envelope pressures. Chen et al.^21^ carried out pre- and post-peak unloading tests under different envelope pressures, different control methods and different unloading rates to elaborate the energy dissipation and energy release characteristics of rocks during deformation and damage, and proposed a new energy discrimination index from the viewpoint of energy and engineering applications.

The above studies only focus on the evolution law of strain energy, such as elastic properties, pre-peak dissipation energy and post-peak fracture energy during the damage of intact rock material specimens, while there are few reports on the study of energy problems related to the damage of coal–rock composite specimens with different inclination angles. By conducting triaxial compression tests on coal rock assemblies with different inclination angles (0°, 5°, 10°, 20°, 30°) under different confining pressures (1 MPa, 2 MPa, 3 MPa), this paper studied the various energy evolution laws during the damage of the assembled coal rocks under different angles and confining pressures, and analyzed the deformation and damage mechanisms. The research results are of great significance to the problems of damaged energy mechanisms, dynamics and disaster mechanisms of combined coal rocks.

## The energy evolution of rock failure processes

**Figure 1:**
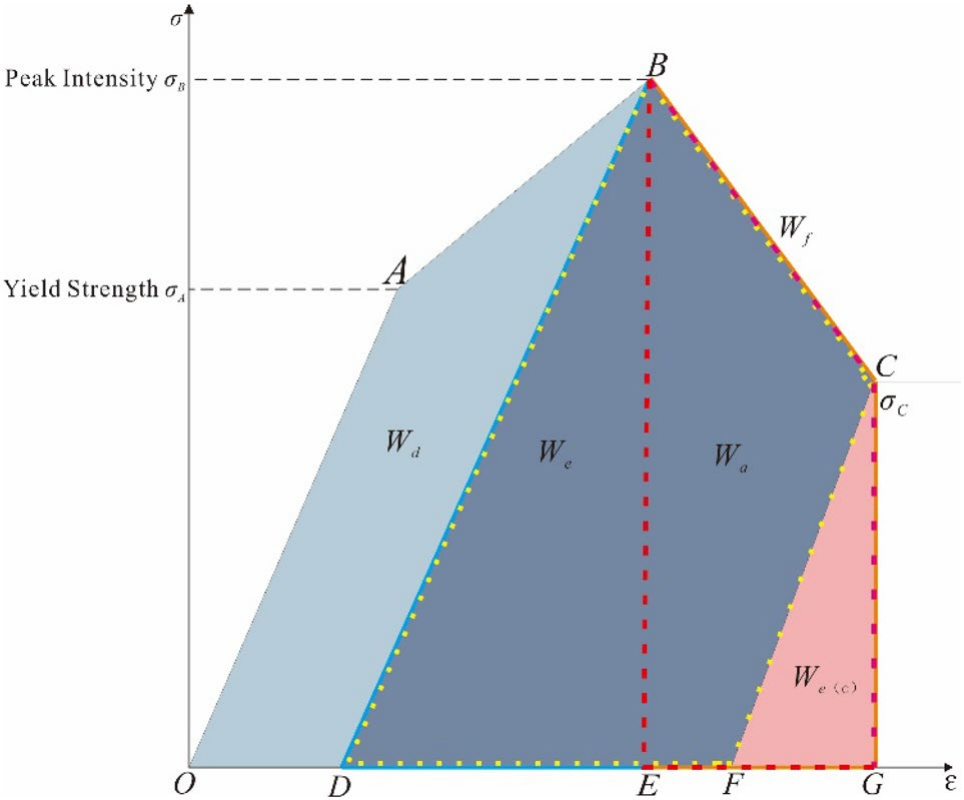
Energy evolution diagram of the whole process of rock failure.

From a macroscopic point of view, rock failure begins with elastic deformation, develops with microcrack expansion, and ends with a complete failure of the rock. Moreover, from the energy point of view, the essence of rock failure is the exchange of internal energy of rock material with external energy, which means, on the one hand, storing the energy transferred from the outside, on the other hand, releasing the internal energy to the outside in various forms. Therefore, the whole energy evolution of rock damage is roughly divided into three stages: energy accumulation (OA section), energy dissipation (AB section), energy transformation and energy release (BC section); as Fig. 1 shown that under the action of axial pressure, the rock is firstly deformed elastically, and the external mechanical energy is accumulated in the form of elastic energy inside the specimen. Finally, macroscopic cracks are formed. The various energies are denoted by *W*_*i*_ with the following meanings: *W*_*d*_ denotes the pre-peak dissipation energy accumulated inside the combination specimen when the peak strength is reached, which means the area in the figure is *S*_*OABD*_; *W*_*e*_ denotes the releasable elastic strain energy, which means the area in the figure is *S*_*BDE*_; *W*_*e*(C)_ denotes the remaining elastic strain energy, which means the area in the figure is *S*_*CFG*_; *W*_*f*_ denotes the incremental post-peak fracture energy, which means the area in the figure is *S*_*DBCF*_; *W*_*a*_ denotes the additional energy required to be provided externally after the peak intensity, that is, the area in the figure is *S*_*BCGE*_.

Assuming that the rock specimen does not exchange heat with the outside world during the physical process, according to the first law of thermodynamics, we can get

1 $$ W_{u} = W_{d} + W_{e} $$

where *W*_*u*_ is the total energy generated by external force work; *W*_*d*_ is the dissipated energy in the pre-peak stage; *W*_*e*_ is the releasable elastic strain energy.

2 $$ W_{u} = \frac{1}{2}\sigma_{A} \varepsilon_{A} + \int_{{\varepsilon_{A} }}^{{\varepsilon_{B} }} {\sigma_{B} } d\varepsilon_{i} $$

3 $$ W_{e} = \frac{1}{2}\sigma_{B} \varepsilon_{B} $$

From Eq. (2) minus Eq. (3) we can get,

4 $$ W_{d} = \frac{1}{2}\sigma_{A} \varepsilon_{A} + \int_{{\varepsilon_{A} }}^{{\varepsilon_{B} }} {\sigma_{B} } d\varepsilon_{i} - \frac{1}{2}\sigma_{B} \varepsilon_{B} $$

The remaining elastic strain energy *W*_*e*(C)_ of the specimen after failure,

5 $$ W_{e(C)} = \frac{1}{2}\sigma_{C} \varepsilon_{A} $$

After the specimen reaches its peak, additional energy *W*_*a*_ is required from the outside,

6 $$ W_{a} = \sigma_{C} \left( {\varepsilon_{C} - \varepsilon_{B} } \right) + \frac{1}{2}\left( {\sigma_{B} - \sigma_{C} } \right)\left( {\varepsilon_{B} - \varepsilon_{C} } \right) $$

Collated to,

7 $$ W_{a} = \frac{1}{2}\left( {\varepsilon_{C} - \varepsilon_{B} } \right)\left( {3\sigma_{C} - \sigma_{B} } \right) $$

Increment *W*_*f*_ of fracture energy after specimen failure,

8 $$ W_{f} = W_{a} + W_{e} - W_{e(C)} $$

## Triaxial compression test of coal–rock combination

### Specimen preparation

**Figure 2:**
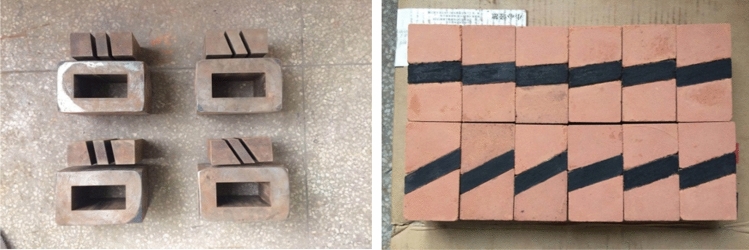
Specimen preparation molds and prepared specimens.

Since this experiment studies the energy evolution characteristics of coal–rock combinations with different inclination angles, it is very difficult to extract complete standard specimens in the field. In order to facilitate the study, it is proposed to use 50 mm×50 mm×100 mm square molds (5 groups of molds with different inclination angles are 0°, 5°, 10°, 20°, 30°) with a sandwich thickness of 20 mm to remold the coal–rock combination specimens. The remodeled rock material was selected from pure natural laterite from a deep excavation in the high mountains of Qingyuan County, Zhejiang Province, and the remodeled coal body was made from pulverized coal from Fuxin open pit mine. The two materials were dried at 105 °C–110 °C for 24 h in a drying oven, and the soil samples and pulverized coal were crushed and milled after removal to screen out particles of 30–40 mesh size. In order to ensure the experimental quality, the ratio of soil sample to water was determined as 10:1. The soil sample and water, coal and water were mixed evenly and placed in the mold, and the upper and lower parts of the test module were pressed in the rigid testing machine with a pressure of 100 kN, and the middle coal powder module was pressed with a pressure of 50 kN. The strength of the upper and lower parts of the test module was higher than that of the middle coal sample module, and the pressed test block was subsequently put into the drying oven at 70 ℃ for 48 h. After being taken out, the specimen is polished, and the three parts are bonded together with adhesive at the coal–rock intersection to make standard specimens with different angles. The two ends of the specimen are carefully ground with a grinding machine and sandpaper to make the upper and lower surfaces parallel to 0.05 mm and the surface flatness within 0.02 mm. Finally, the specimens were tested for ultrasonic wave velocity, and nine groups (15 specimens in each group) of coal–rock combination specimens with little difference in wave velocity were taken as specimens. The experimental molds and the prepared specimens are shown in Fig. 2 below.

### Test equipment and methods

**Figure 3:**
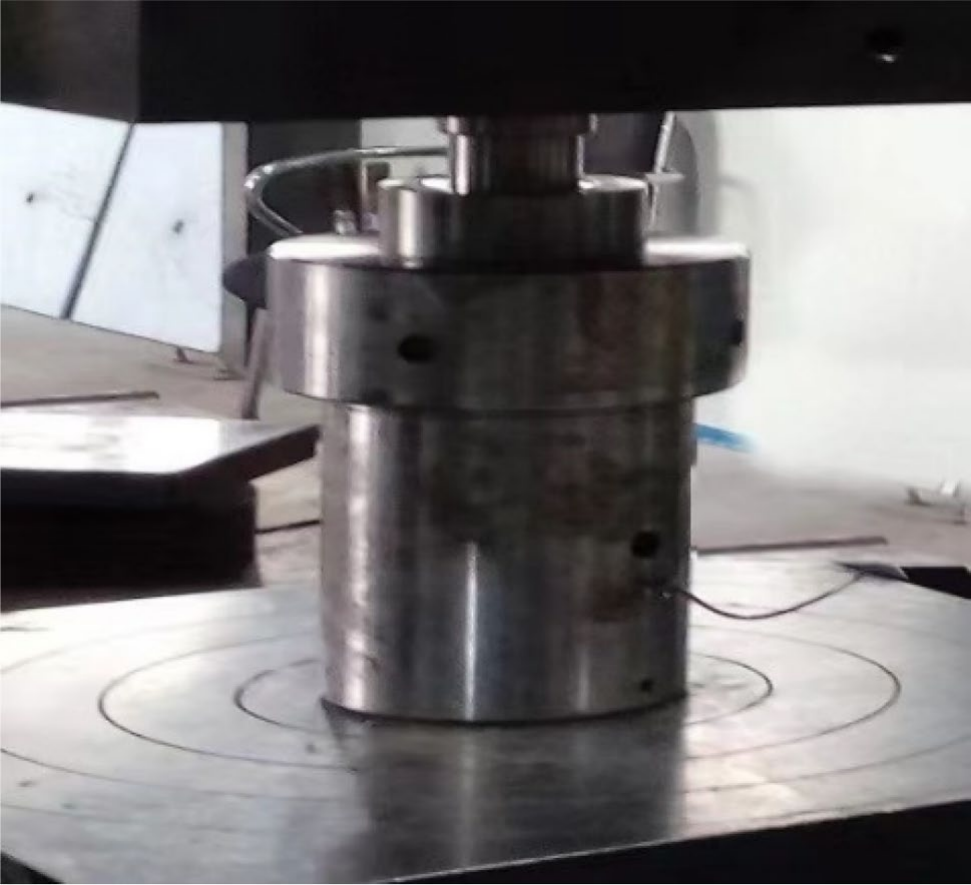
MTS815 testing machine.

This test was completed on the MTS815 testing machine, and three groups of each rock-coal–rock combination with different inclination angles (0°, 5°, 10°, 20°, 30°) were carried out to carry out triaxial compression tests under different confining pressures (1 MPa, 2 MPa, 3 MPa), as shown in Fig. 3. The above tests were repeated, and the mechanical parameters of the coal–rock combination were obtained by collating the test data, as shown in Table 1.

**Table 1:** Physical and mechanical parameters of coal–rock combined.

Specimen number	Combined tilt angle (°)	*σ*_3_ (MPa)	*E*_*ave*_ (GPa)	*σ*_1_ (MPa)	*ε*_*p*_	Failure mode and breaking angle (°)
0-1	0	1	0.345	7.551	0.028	Inclined shear damage, 61.579
0-2		2	0.329	12.966	0.041	Inclined shear damage, 61.558
0-3		3	0.440	14.380	0.054	Inclined shear damage, 61.614
5-1	5	1	0.365	7.181	0.026	Inclined shear damage, 59.214
5-2		2	0.391	11.787	0.037	Inclined shear damage, 59.413
5-3		3	0.444	14.345	0.052	Inclined shear damage, 59.259
10-1	10	1	0.364	7.076	0.025	Inclined shear damage, 58.114
10-2		2	0.390	10.870	0.036	Inclined shear damage, 58.471
10-3		3	0.405	13.693	0.048	Inclined shear damage, 58.104
20-1	20	1	0.377	6.535	0.024	Tensile and diagonal shear damage, 57.631
20-2		2	0.385	10.447	0.035	Tensile and diagonal shear damage, 57.236
20-3		3	0.425	12.236	0.043	Tensile and diagonal shear damage, 57.518
30-1	30	1	0.331	5.379	0.019	Tensile and diagonal shear damage, 56.820
30-2		2	0.347	9.215	0.029	Tensile and diagonal shear damage, 56.744
30-3		3	0.476	10.057	0.033	Tensile and diagonal shear damage, 56.438
Rock-1	Homogeneous rock mass	1	0.322	8.084	0.029	Shear damage, 64.662
Rock-2		2	0.354	13.097	0.045	Shear damage, 64.346
Rock-3		3	0.416	17.085	0.055	Shear damage, 64.501
Coal body 1	Homogeneous coal body	1	0.336	4.764	0.016	Tensile and shear damage, 47.576
Coal body 2		2	0.353	7.097	0.022	Tensile and shear damage, 47.372
Coal body 3		3	0.478	8.392	0.028	Tensile and shear damage, 47.519

### Test results and analysis

**Figure 4:**
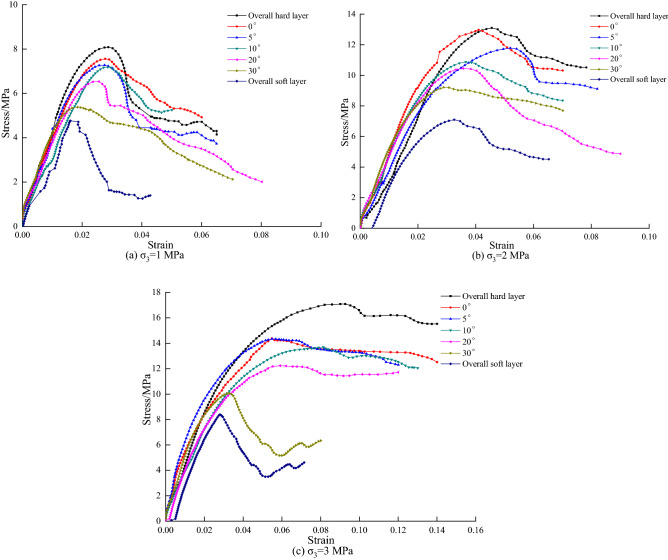
Stress–strain curve of coal–rock combined.

From Fig. 4, it can be seen that the peak intensity of the coal–rock combination with different dip angles is located between coal–rock monoliths, and the peak stress of both coal–rock combination and coal–rock monoliths increases with the increase of confining pressure. Among the rock, coal–rock combination and coal body, the rock body can enter the compression-density stage firstly, followed by the coal–rock combination and the coal body; the speed of entering the compression-density stage becomes slower as the inclination angle increases in the coal–rock combination. The duration of the elastic deformation stage is the opposite of the compression stage, that is to say, the quicker the compression stage is entered, the longer the duration of the elastic deformation stage will be.

**Figure 5:**
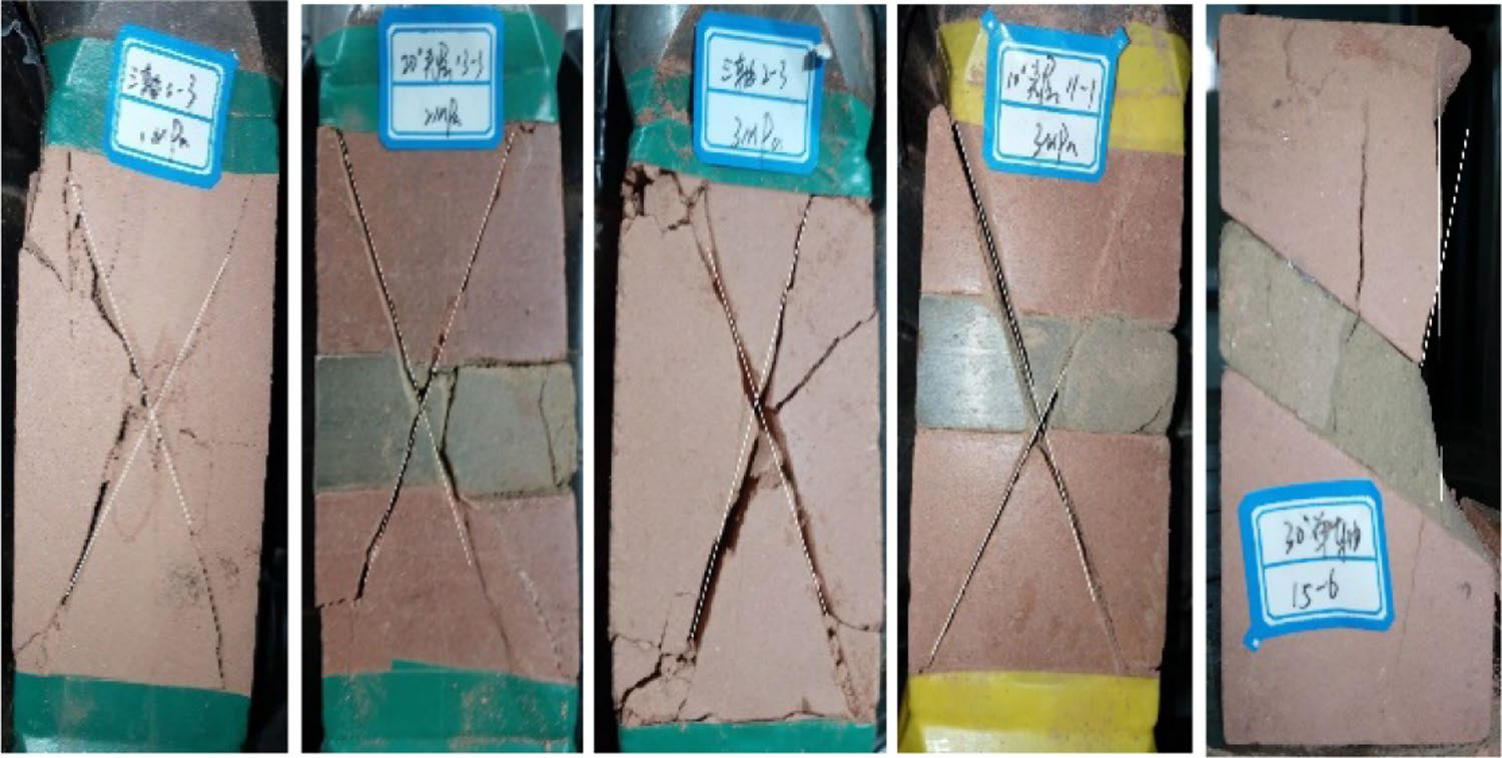
Typical failure patterns of some samples under different confining pressures and different angle.

According to Table 1 and Fig. 5, it can be known that the coal–rock monoliths without inclusions show similar damage characteristics in the deformation and damage process, and they both show typical X-shaped conjugate shear damage, in which the average breaking angle of the rock body is 64.503°, the average breaking angle of the coal body is 47.489°, and the breaking angle decreases gradually with the increase of the strength of the coal–rock body. Since the coal–rock combination is not a complete rock body with a jointing surface, the coal body itself has a large number of joints, fissures and weak faces of joints, fissures and weak faces, which will be the first to be destabilized when triaxial compression occurs; thus the combination body damage is mainly oblique shear damage. When the combined coal rock body dip angle φ ≥ 10°, the combined specimen appears to be damaged by the combination of oblique shear damage and tensile damage. With the increase of the inclination angle of the coal–rock combination, the breaking angle also increases, and the rate of increase also gradually decreases, indicating that the angle of the joint surface seriously affects the damage mode and the breaking degree of the coal–rock combination.

## Energy evolution law of coal–rock combination failure process

### Simplifying the whole stress–strain curve

**Figure 6:**
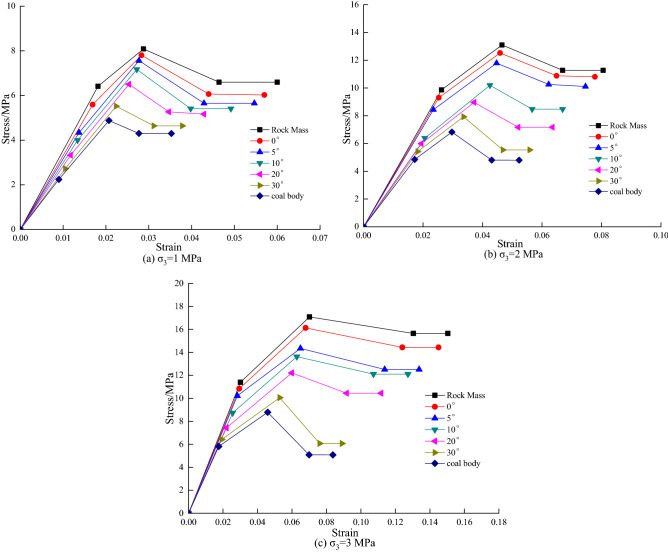
Simplified diagram of full stress–strain curve.

After the yield stress *σ*_A_ is reached, with the increasing external load, the rock specimen enters the plastic yield stage (crack non-stable expansion stage), and the microcrack inside the specimen is formed and starts to expand, while some energy is dissipated and the stress–strain curve is parabolic. As the stress gradually approaches the peak strength *σ*_B_, the microcracks increase rapidly and eventually converge to form macroscopic fracture cracks. According to the first law of thermodynamics, assuming that there is no heat exchange between the failure process of rock materials and the outside world, the full stress–strain curve of the triaxial compression test of coal–rock combination at different dip angles is simplified to the ideal curve form as Fig. 6 according to the time of rock failure crack generation.

### Energy evolution law of coal–rock combination

Using Eqs. (3) and (4), Eqs. (7) and (8), the different energy values in the simplified stress–strain diagram are obtained, and the statistics are shown in Table 2 below.

**Table 2:** Different energy and index statistics table.

Inclination (°)	Confining pressure (MPa)	*W*_*d*_	*W*_*e*_	*W*_e(*C*)_	*W*_*f*_	*W*_*a*_	*B*_pre1_	*B*_pre2_	*B*_post1_	*B*_post2_
0	1	0.042	0.093	0.061	4.184	0.130	1.025	0.025	130.75	3.697
	2	0.134	0.228	0.169	16.329	0.249	2.354	1.354	276.763	4.220
	3	0.356	0.386	0.324	25.857	0.986	2.664	1.664	417.048	15.903
5	1	0.022	0.091	0.051	4.030	0.100	1.370	0.370	100.750	2.500
	2	0.119	0.193	0.146	6.170	0.190	2.448	1.266	131.276	4.343
	3	0.309	0.285	0.217	18.660	0.660	2.797	1.797	274.412	9.706
10	1	0.005	0.086	0.049	3.522	0.079	1.081	0.081	95.189	1.766
	2	0.019	0.167	0.116	5.413	0.133	1.188	0.188	106.137	2.608
	3	0.056	0.271	0.215	16.986	0.572	1.373	0.373	303.321	10.214
20	1	0.004	0.075	0.049	2.531	0.055	1.074	0.074	97.340	2.145
	2	0.012	0.130	0.083	4.921	0.120	1.167	0.167	104.702	2.553
	3	0.057	0.221	0.162	11.443	0.361	1.413	0.413	199.949	6.119
30	1	0.002	0.059	0.041	2.083	0.045	1.044	0.044	51.157	1.388
	2	0.009	0.105	0.051	4.309	0.089	1.161	0.161	79.796	1.648
	3	0.041	0.151	0.094	6.788	0.291	1.461	0.461	119.088	5.105

Fitting the data in Table 2 with nonlinearity, the relationship between energy and the inclination of the coal–rock combination is obtained as

9 $$ W = A{ \cdot }\exp \left( {{{ - \varphi } \mathord{\left/ {\vphantom {{ - \varphi } x}} \right. \kern-\nulldelimiterspace} x}} \right) + B $$

where *W* denotes the energy; *φ* denotes the inclination angle of the coal–rock combination; *A*, *B* and *C* are the fitting coefficients.

**Figure 7:**
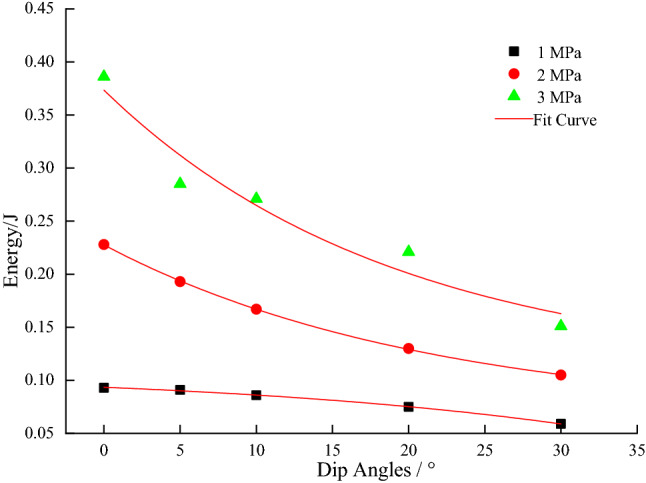
The relationship between peak front dissipation energy and angle.

**Figure 8:**
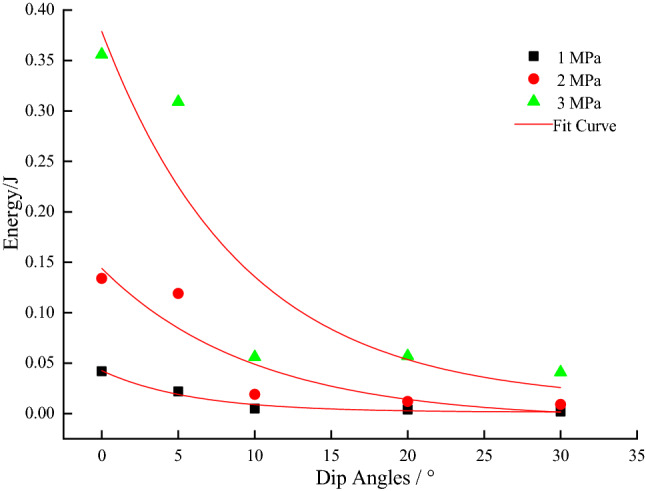
The relationship between the releasable elastic strain energy and the inclination angle.

**Figure 9:**
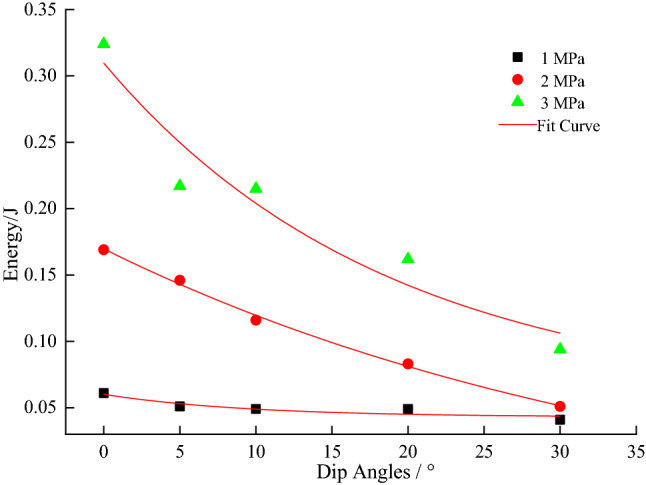
Residual elastic strain energy as a function of inclination angle.

**Figure 10:**
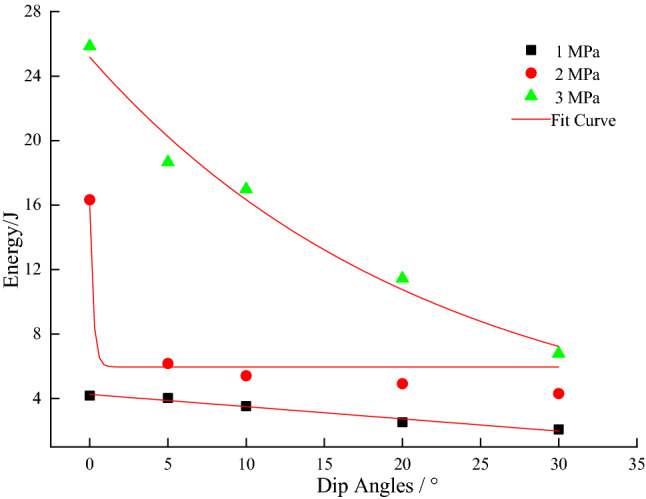
Fracture energy increment of post-peak rocks as a function of dip angle.

**Figure 11:**
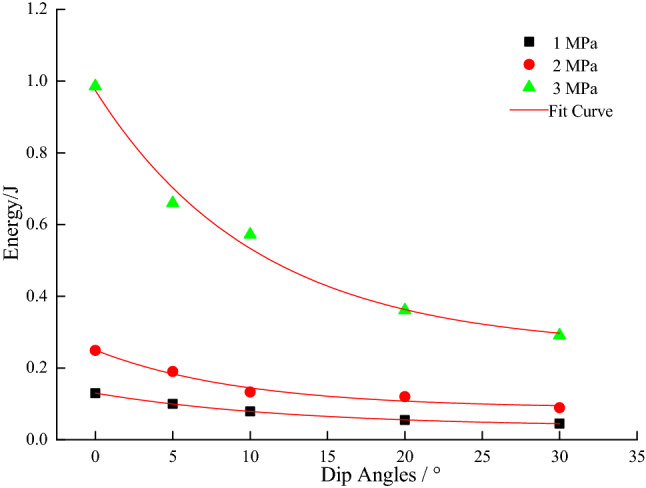
Need for additional energy from outside after peak intensity.

From Figs. 7, 8, 9, 10 and 11, it can be seen that various energies (pre-peak dissipated energy, releasable elastic strain energy, remaining elastic strain energy, post-peak rock fracture energy increment, and additional energy required from outside after peak intensity) are distributed exponentially as a function of the gradual increase in the dip angle of the coal–rock combination with a non-linear decrease at constant confining pressure. When the confining pressure is at 1 MPa, the change of various energies is small. However, when the confining pressure exceeds 3Mpa, the energy decreases linearly, reflecting the strong energy sensitivity to the confining pressure. When the dip angle of the coal–rock combination is constant, the energy decreases as a negative exponential function with the increase of the dip angle of the coal–rock combination. The rate of energy decrease is the largest when the inclination angle increases from 0° to 5°, and its decrease is less obvious from 5° to 30°. By comparing various energies, the incremental rock fracture energy after the peak is much higher than other energies because external mechanical energy is mainly converted into incremental rock fracture energy after the peak.

Based on the two pre-peak exponents *B*_pre1_, *B*_pre2_ and two post-peak exponents *B*_post1_, *B*_post2_ proposed by Zhang Jun^22^ to characterize rock brittleness, the characteristics of the four exponents of coal–rock combinations under different dip angles and confining pressures were studied;

10 $$ B_{{{\text{pre}} 1}} = \frac{{W_{d}^{*} }}{{W_{e} - W_{e\left( A \right)} }} $$

11 $$ B_{{{\text{pre}} 2}} = \frac{{W_{d} }}{{W_{e} - W_{e\left( A \right)} }} $$

12 $$ B_{{{\text{p}} {\text{ost}} 1}} = \frac{{W_{f} }}{{W_{e} - W_{e\left( C \right)} }} $$

13 $$ B_{{{\text{p}} {\text{ost}}2}} = \frac{{W_{a} }}{{W_{e} - W_{e\left( C \right)} }} $$

where *B*_pre1_ is used to measure the plastic yield degree in the pre-peak stage, *B*_pre2_ is used to measure the energy dissipation level in the pre-peak stage, *B*_post1_ is used to represent the rock crack propagation ability in the post-peak stage, and *B*_post2_ is used to represent the ability of the material to sustain fracture by itself. *W*_*e*_ – *W*_*e*(A)_ is the elastic energy increment in the yielding stage, *W*_d_^*^ = *W*_*d*_ + *W*_*e*_ − *W*_*e*(A)_ is the total energy accumulated in the yielding stage; *W*_*e*_ − *W*_*e*(C)_ is the unloading elastic energy.

**Figure 12:**
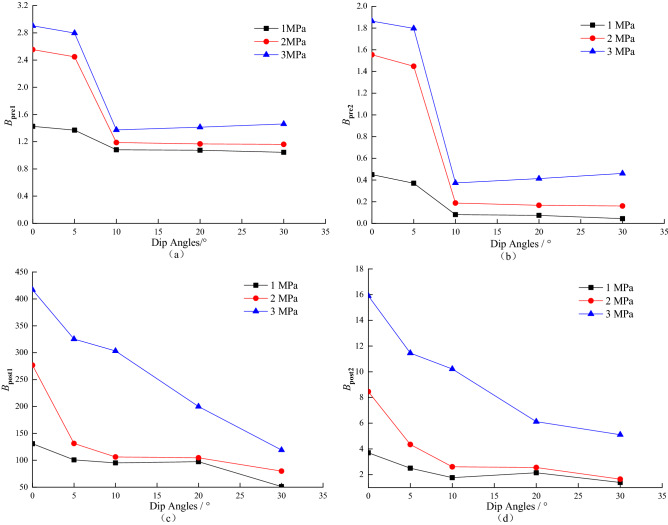
The variation curves of each index under different circumferential pressure and inclination angle.

According to the *B*_pre1_, *B*_pre2_, *B*_post1_, and *B*_post2_, variation curves characterizing the rock brittleness index under different circumferential pressures obtained in Table 2; as shown in Fig. 12, with the increase of coal–rock combination dip angle, the *B*_pre1_, *B*_pre2_, *B*_post1_, and *B*_post2_ values gradually decrease, and the plastic yielding degree and energy dissipation level in the pre-peak stage gradually decrease. The faster the crack expansion and fracture rate in the process, the more easily the rock is damaged. At the same dip angle, the values of confining pressure and brittleness indexes *B*_pre1_, *B*_pre2_, *B*_post1_, and *B*_post2_ are positively correlated, indicating that the confining pressure has a greater influence on the crack expansion ability and self-sustaining fracture ability of the post-peak rock.

### Deformation and damage mechanism of coal–rock combination

The failure process of the coal rock body is essentially the mechanical equilibrium state of internal and external physical changes when stress reaches the limit of failure; the elastic energy accumulated in the system is greater than the energy consumed due to the system failure, the excess elastic energy released to the outside and lead to the failure of the coal rock body. In the pre-peak stage, part of the energy generated by the external force is consumed due to the damage and plastic deformation of the rock, and the other part is converted into elastic strain energy stored in the rock. With the increase in the dip angle of the coal–rock combination, the form of damage changes from the small dip angle of the bevel shear damage to the larger dip angle of the bevel shear and tensile combination of damage, and the energy required for damage is also gradually reduced. When the peak is reached, the elastic strain energy stored before the peak of the rock is not enough to induce complete failure of the rock, which must rely on additional energy from the outside, so that the complete failure of the rock depends on the elastic strain energy that can be released and the additional energy provided by the outside. In the post-peak stage, the elastic strain energy stored at the time of damage is released in large quantities, and the elastic strain energy storage capacity is also reduced at this stage due to the crack expansion and structural recombination of the rock during rupture, which results in a sudden decrease and then a small increase in the elastic strain energy. The smaller the inclination angle or larger the confining pressure, the smaller the decrease of elastic strain energy at the peak damage, indicating that different inclination angles and confining pressures restrict the damage of coal–rock combination specimens. The fracture energy increment occurs when the coal–rock combination is damaged, and the increment accounts for the largest ratio of various energies in the stress–strain curve of the coal–rock combination, and the size of the fracture energy increment is an important basis for judging the brittleness of the rock.

## Conclusion

(1) The peak strength of the coal–rock combination lies between the coal–rock monolith, and the combination's yield strength decreases as the combination's inclination angle increases, showing a damage pattern of a combination of oblique shear damage and tensile damage during the damage process. In contrast, the coal–rock monolith is mainly X-shaped conjugate shear damage.

(2) It is revealed that during the failure process of coal–rock combination, with the increase of angle, the plastic yielding degree and energy dissipation level gradually decreases, the crack expansion and fracture speed is accelerated, and the failure is very easy to occur, and it also shows the law that the confining pressure is more sensitive to the crack expansion ability and self-sustaining fracture ability of the post-peak rock.

(3) From the Angle of energy, the whole rock stress—strain curve is the external performance, to its internal energy state transitions from elastic deformation, and microcrack evolution, until the destruction of the entire process, always accompanied by rock materials and energy exchange of the outside world, on the one hand, storage of energy from the outside, on the other hand, and release energy to the world and with a variety of forms to maintain energy balance.

(4) The deformation and failure process of rock is essentially the whole process of energy dissipation and energy release. The elastic energy accumulated in rock is the main source force of rock fracture, and the degree of pre-peak energy dissipation determines the level of post-peak fracture energy. The essence of brittle rock damage is the dynamic instability phenomenon caused by the accumulation of high energy in the pre-peak phase and the rapid release of energy in the post-peak phase. The pre-peak dissipation energy and post-peak fracture energy are the essential factors that determine brittle fracture in rocks; the smaller the ratio of pre-peak dissipation energy to post-peak fracture energy, the more brittle the rock is when the elastic energy accumulated inside the rock is specific. The inclination angle of the coal–rock combination obeys a negative exponential function distribution with the pre-peak dissipation energy, the releasable elastic strain energy, the remaining elastic strain energy after rock failure, the incremental rock fracture energy after the peak, and the additional energy required to be provided externally after the peak strength.

(5) When the inclination angle of the coal–rock combination is constant, the confining pressure and various energies are positively correlated, where the magnitude of the confining pressure affects the energy storage capacity of the rock; in the low confining pressure stage, that is, when the confining pressure is less than 3 MPa, the various energies change little with the increase of the inclination angle of the coal–rock combination. However, when the confining pressure exceeds 3 MPa, the energy decreases linearly, reflecting the strong sensitivity to the confining pressure. When the inclination angle of the coal–rock combination is certain, the energy decreases with the increase of the inclination angle of the coal–rock combination as a negative exponential function.

(6) By calculating the values of the brittleness indexes of the characterized coal rock combination, it is concluded that the values of the brittleness indexes *B*_pre1_、 *B*_pre2_、 *B*_post1_ and *B*_post2_ gradually decrease with the increase of the inclination angle of the coal rock combination; at the same inclination angle, the values of the confining pressure and the brittleness indexes *B*_pre1_、 *B*_pre2_、 *B*_post1_ and *B*_post2_ are positively correlated.

## Data Availability

The data used to support the findings of this study are included within the article.
